# Navigating lipid droplet proteins – part I: ER targeting routes of lipid droplet-destined membrane proteins

**DOI:** 10.1042/BST20253051

**Published:** 2025-10-29

**Authors:** Louisa Magdalena Krauß, Bianca Schrul

**Affiliations:** 1 Medical Biochemistry and Molecular Biology, Center for Molecular Signaling (PZMS), Faculty of Medicine, Saarland University, Homburg/Saar, 66421, Germany; 2 Center for Biophysics, Saarland University, Germany

**Keywords:** endoplasmic reticulum, lipid droplets, membrane proteins, membranes, organelles, cellular targeting, transmembrane proteins

## Abstract

Lipid droplets (LDs) are cytosolic lipid storage organelles that derive from the endoplasmic reticulum (ER). Their biogenesis and function are essential for maintaining cellular lipid homeostasis and require a spatiotemporally co-ordinated recruitment of specific membrane proteins to the LD surface. Many LD-destined proteins are inserted into the ER phospholipid bilayer in a monotopic hairpin topology before they can partition to the LD monolayer. About a third of all cellular proteins enter the ER during their biogenesis, either as ER-resident or as secretory proteins. Decades of research have provided a solid understanding of which molecular machineries ensure ER targeting fidelity of transmembrane-spanning proteins. The molecular mechanisms underlying the biogenesis of LD-destined monotopic proteins, however, are only beginning to emerge. In this first part of the bipartite review ‘Navigating lipid droplet proteins,’ we provide an overview of the general principles underlying protein targeting to the ER. We highlight recent advances and current challenges regarding the specific mechanisms for LD-destined proteins and discuss their physiological implications. The molecular mechanisms underlying the subsequent ER-to-LD protein partitioning are at the heart of the second part of this bipartite review.

## 1. Introduction

### 1.1 LD-localized proteins are key for cellular metabolism

Lipid droplets (LDs) are evolutionarily conserved and ubiquitous organelles that play a pivotal role in cellular lipid and energy metabolism. They dynamically store and mobilize neutral lipids in response to metabolic signaling cues and thereby serve as reservoirs for ATP production or the synthesis of membrane precursors and lipidic signaling molecules. LDs also function as lipid buffers, limiting toxicity from excessive free fatty acids [1]. This multifunctionality of LDs depends on specific proteins associated with their surface [2–7], including metabolic enzymes involved in the synthesis or breakdown of neutral lipids, tethering proteins that mediate organelle contacts for transfer and metabolization of fatty acids, as well as factors supporting LD structural integrity. Precise spatially and temporally regulated delivery of specific proteins to the LD surface is fundamental for maintaining cellular homeostasis, as aberrations are implicated in various metabolic pathologies such as obesity, cardiovascular disorders, and cancer [8–10].

**Figure 1:**
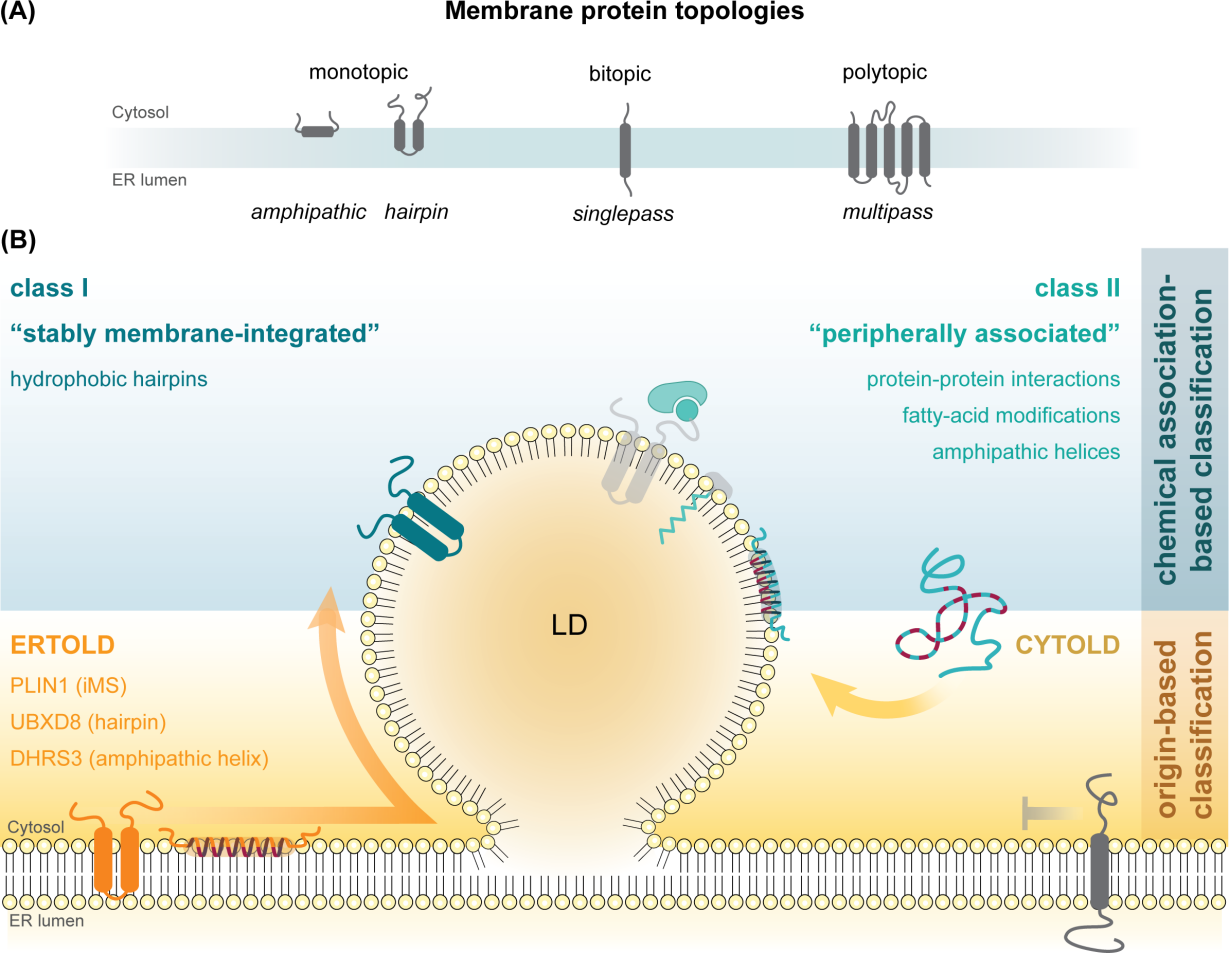
Classification of LD proteins. (**A**) Endoplasmic reticulum (ER) membrane proteins are categorized as monotopic, bitopic, or polytopic based on the orientation of their termini toward the cytosol or the ER lumen. Bi- and polytopic membrane proteins span the ER bilayer with one or multiple transmembrane regions, respectively, whereas monotopic membrane proteins embed into the membrane using amphipathic helices or hydrophobic hairpin regions, without exposing soluble domains into the lumen. (**B**) Lipid droplet (LD) proteins can be classified by the type of chemical interaction with the phospholipid monolayer (top) or based on their subcellular origination (bottom), targeting the LD surface either directly from the cytosol (CYTOLD) or from the ER membrane (ERTOLD). While protein association with the LD monolayer can be mediated by protein-protein interactions, fatty acid modifications, amphipathic helices, or hydrophobic hairpin regions, some LD proteins possess multiple distinct LD-targeting motifs, as exemplified by ANKRD22, which harbors a hydrophobic hairpin region with a central proline alongside an N-myristoylation motif that positively affects its LD localization [13]. As biochemical characterization of LD proteins expands, novel LD-binding motifs continue to emerge. For instance, PLIN1 features an alternative, atypical integral membrane segment (iMS) for ER and LD localization. While similar in length to other known hydrophobic hairpin regions and sharing key characteristics such as a central proline residue, the PLIN1 iMS is markedly less hydrophobic [14]. Proteins that associate with LDs by interaction with LD-resident proteins can, in principle, also follow the ERTOLD route, despite originating from the cytosol. This occurs when their interaction partner is initially ER-localized, causing them to co-partition to the LD surface. An example is the AAA-ATPase VCP/p97, which binds to the ERTOLD protein UBXD8 in both membrane environments [15]. PLIN, perilipin; DHRS3, short-chain dehydrogenase/reductase 3; UBXD8, UBX domain-containing protein 8; ANKRD22, ankyrin repeat domain-containing protein 22; VCP, valosin-containing protein.

LDs originate from the endoplasmic reticulum (ER), where neutral lipids are synthesized and accumulate between the phospholipid leaflets of the ER bilayer membrane, leading to LD growth and budding toward the cytosol [reviewed in detail in [1,11,12] (Figure 1)]. Mature LDs consist of a hydrophobic neutral lipid core encircled by a phospholipid monolayer, a unique architecture in the cell that directly influences protein interactions with LDs: the hydrophobic nature of the neutral lipid core precludes direct protein incorporation, restricting associations to the phospholipid monolayer. At this interface, proteins either bind peripherally or embed stably via hydrophobic segments. Hydrophilic protein domains, however, must remain oriented toward the cytosol, enforcing a monotopic membrane topology [16–18] (Figure 1A).

### 1.2 LD protein classification – an evolving framework

Historically, LD proteins have been classified based on their chemical interaction with the LD monolayer (Figure 1B) [19]. Class I proteins were considered to target the LD monolayer from the ER membrane and to stably integrate into both membranes with the aid of hydrophobic regions that consist of two alpha-helical membrane segments separated by a kink region that frequently includes a central proline. This results in a re-entrant helix-turn-helix conformation, which was initially conceptualized as a hairpin-shaped structure partially embedded into the ER membrane. Such a conformation would allow coexistence of these proteins in both ER bilayers and LD monolayers and enable passive lateral diffusion between both compartments (for details, see part II of this bipartite review, [20]). In contrast, class II proteins were considered to associate with the LD monolayer only peripherally employing amphipathic helices or lipid anchors, or via protein–protein interactions.

When this classification system was first established [19], hardly any LD proteins had been characterized biochemically or structurally, and only subsequent investigations revealed that several proteins targeting the LD monolayer from the ER are not hairpin proteins but associate with both membranes via interfacial amphipathic helices [21]. Thus, a more recently proposed ‘origin-based classification’ of LD proteins (Figure 1B) appears more appropriate. Here, proteins that target the LD monolayer from the ER membrane, irrespective of how they chemically interact with the membranes, are referred to as ERTOLD proteins, whereas proteins that bind to the LD surface from the cytosol are called CYTOLD proteins [22]. Both classes undergo a multi-step biogenesis process to ultimately reach their destination at the LD surface, with a central challenge being the adoption of a monotopic membrane topology compatible with the unique LD architecture. For ERTOLD proteins, this includes their synthesis, ER targeting, and membrane insertion, followed by partitioning to the LD monolayer membrane. ER-to-LD partitioning can occur either early during LD formation when proteins traverse the ER-resident LD biogenesis machinery or later during LD maturation via ER-LD membrane continuities [23]. The processes driving differential protein recruitment from the ER to LDs are discussed in part II of this bipartite review ‘Navigating Lipid Droplet Proteins’[20].

Here, we discuss recent findings regarding the molecular mechanisms underlying the initial ER targeting of LD-destined proteins and highlight emerging insights into its plasticity, structural determinants for pathway selection, and topological rearrangements occurring during membrane insertion. We furthermore outline unresolved questions regarding modulated ER targeting in response to physiological demands and the relevance of dedicated ER targeting machinery in coordinating organelle biogenesis from the ER.

## 2. Multiple pathways – one destination: ER targeting of LD-destined proteins

Decades of research have fundamentally advanced our understanding of how cells ensure faithful ER targeting of ER-resident or secretory proteins, revealing highly conserved yet increasingly complex pathways [24–29]. The ongoing identification of regulatory factors underscores that key mechanistic layers of ER targeting remain to be elucidated, which may become particularly relevant for specialized cases, such as the biogenesis of hairpin-containing ERTOLD proteins.

**Figure 2:**
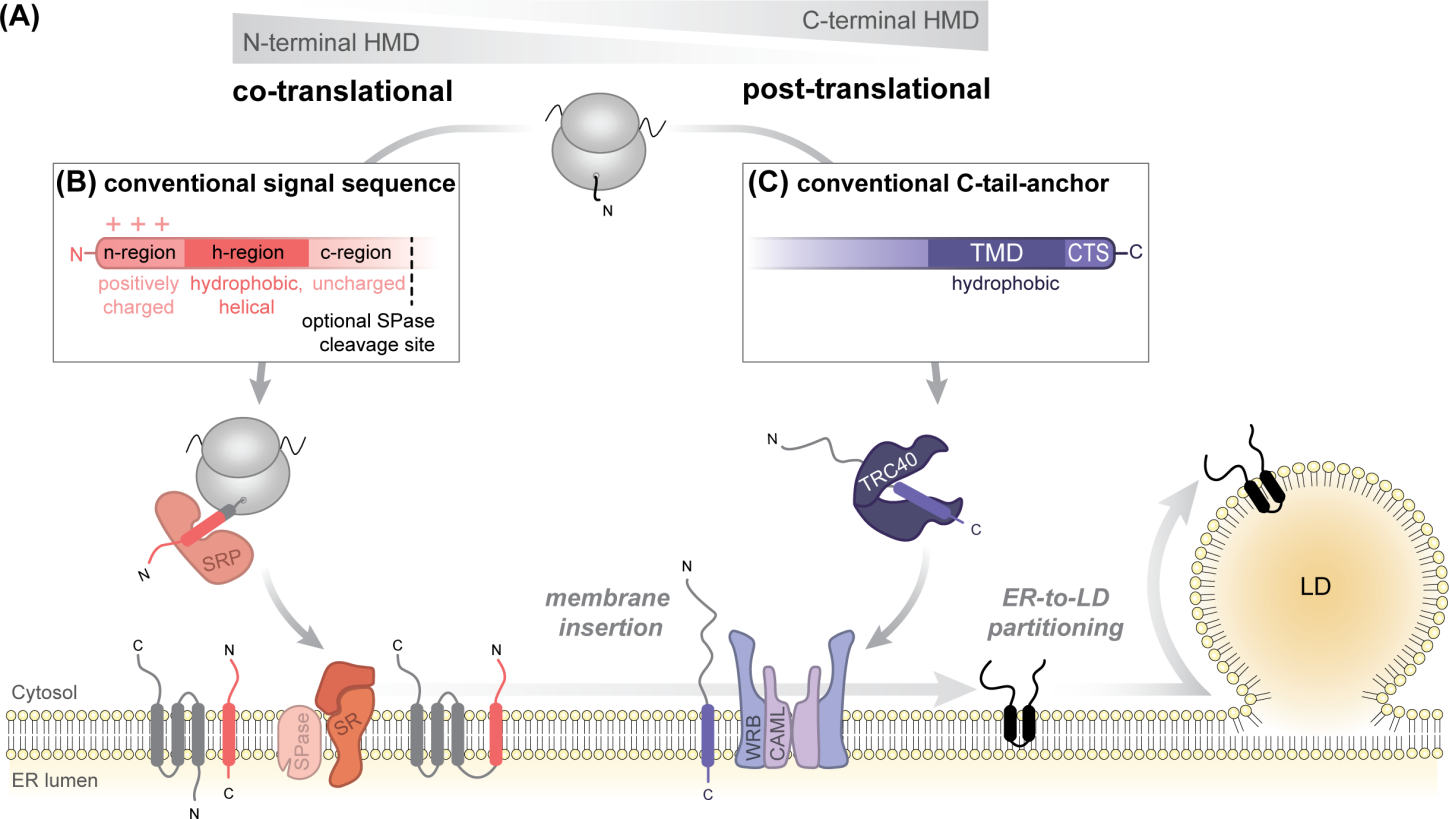
Canonical ER targeting and membrane insertion routes for signal sequence- or tail anchor-comprising transmembrane proteins. (**A**) Endoplasmic reticulum (ER) targeting information of newly synthesized proteins is usually encoded within their hydrophobic membrane domains (HMDs) that are integrated into the membrane. Left: The signal recognition particle (SRP) pathway mediates the co-translational ER targeting of proteins with N-terminal signal sequences or signal anchor sequences. SRP binds to the emerging signal sequence and delivers the ribosome-nascent chain complex to the ER-resident SRP receptor (SR) for protein handover to membrane insertases or protein-conducting channels. Right: The transmembrane recognition complex (TRC/GET) pathway facilitates post-translational ER targeting of tail-anchored (TA) proteins, which contain a transmembrane domain (TMD) near their extreme C-terminus. TRC40 (Get3 in yeast) binds to the TMD and guides it to the ER-resident WRB/CAML (Get1/Get2 in yeast) receptor complex for insertion. ERTOLD proteins do not contain conventional signal sequences or tail-anchor sequences, yet they must be correctly targeted to the ER membrane and inserted in a monotopic topology enabling them to subsequently partition to the lipid droplet (LD) surface. (**B**) Outline of a conventional N-terminal signal sequence facilitating SRP-mediated ER targeting. It comprises three domains: a hydrophilic, positively charged n-domain (3-100 residues); a hydrophobic h-domain (7–15 residues, alpha-helix-prone) and a polar, uncharged c-domain (3–10 residues), which may contain a consensus motif for cleavage by signal peptidase (SPase) [24,31,32]. (**C**) Outline of a conventional C-tail-anchor targeted to the ER via the TRC pathway. A single TMD is positioned within the last 50 residues at the C-terminus, flanked by a C-terminal segment (CTS) that may contain positively charged residues [33]. TMD hydrophobicity, length, and the net charge of flanking regions influence the specificity of TA protein targeting to the ER or other organellar membranes [34,35].

To achieve precise targeting to the ER, cells employ intricate machineries that operate following a shared underlying principle: a soluble cytosolic targeting factor recognizes specific intrinsic signal sequences in the cargo protein and directs it to a designated ER membrane-bound receptor, which subsequently transfers it to the respective protein-conducting channel or insertase, depending on the cargo’s topology and biogenesis route [24,29,30]. The delivery to the ER membrane may either occur during protein synthesis (co-translationally) or after the protein has been fully synthesized and released from the ribosome (post-translationally). Two primary pathways have been described, which can facilitate the targeting of transmembrane-spanning membrane proteins into the ER membrane: the co-translational signal recognition particle (SRP) system and the post-translational transmembrane recognition complex (TRC) system [termed guided entry of tail-anchored (GET) in yeast] (Figure 2).

### 2.1 SRP-mediated ER targeting of LD-destined proteins

Most ER-targeted membrane proteins utilize the co-translational SRP system for ER insertion. Since the founding discovery by Blobel and colleagues in the 1980s [36–38], we nowadays have a detailed understanding of the core principles underlying this pathway [24,27,28]. In brief, cytosolic SRP recognizes N-terminal signal sequences as soon as they are emerging from the ribosomal exit tunnel during translation. It then directs the ribosome-nascent chain complex to the ER membrane, where it binds to its cognate heterodimeric SRP receptor (SR) (Figure 2). Insertion of the nascent polypeptide chain into the membrane is usually mediated by the Sec61 translocon complex, which facilitates the lateral insertion of the transmembrane-spanning segments into the ER bilayer and translocation of soluble domains across the membrane. Charged amino acids flanking the transmembrane domain (TMD) determine the membrane topology of bitopic and polytopic membrane-spanning proteins, generally positioning positive charges on the cytosolic side, in accordance with the ‘positive inside rule’ [39]. Signal sequences are considered the key determinants for fidelity in the co-translational, ER-selective membrane targeting (Figure 2B). They may be cleaved off from the preproteins by signal peptidase during translation or function as signal-anchor sequences as part of the mature proteins.

ERTOLD proteins, however, lack cleavable N-terminal signal sequences and are heterogenous regarding the physicochemical properties and position of their hydrophobic regions. Unlike for transmembrane proteins, which fully span the bilayer membrane, no soluble protein domains need to be translocated across the ER membrane for monotopic proteins, questioning the necessity for a protein-conducting channel or translocase activity. Instead, membrane integration of monotopic proteins may rather require an insertase function, which also provides specificity for select ER targeting and prevents mistargeting to other organelles. Yet, plant oleosins, the first LD-localized proteins studied with respect to their ER insertion pathways, depend on the SRP system and the Sec61 translocon for their co-translational ER membrane insertion [40,41]. Similarly, caveolin-1, a mammalian protein predominantly localizing to caveolae in the plasma membrane but also associating with LDs under certain conditions, utilizes the same molecular machinery for ER insertion [42–44].

#### 2.1.1 ER-resident machinery for co-translational insertion of LD-destined proteins: Sec61 meets EMC

**Figure 3:**
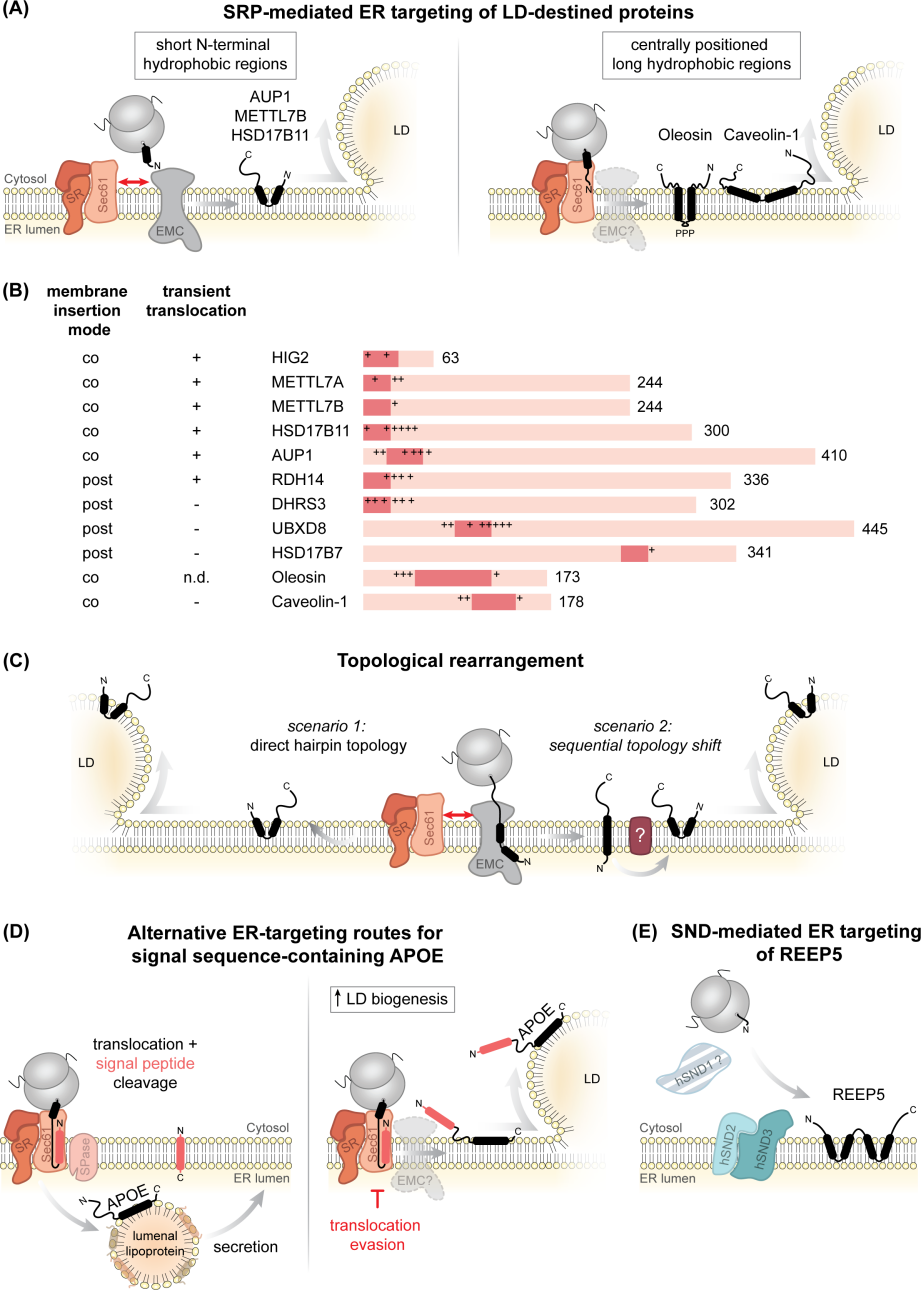
Co-translational ER membrane insertion of ERTOLD proteins. (**A**) Several ERTOLD proteins utilize the signal recognition particle (SRP) pathway to reach the endoplasmic reticulum (ER) membrane. Left: ERTOLD proteins with short N-terminal hydrophobic regions (e.g. AUP1, METTL7B, HSD17B11) insert via Sec61 and the ER membrane complex (EMC), transiently exposing their N-termini to the ER lumen. Right: ERTOLD proteins with more centrally positioned long hydrophobic regions (e.g. caveolin-1 and plant oleosin) also rely on SRP and Sec61, though EMC involvement in their membrane insertion remains unclear. (**B**) Domain organization of the ERTOLD proteins HIG2 [46], METTL7A/B [47–49], HSD17B11 [50], AUP1 [17,51], RDH14 [21], DHRS3 [21,52], UBXD8 [15,47,53,54], HSD17B7 [45], oleosin [41,55–57], and caveolin-1 [42,58], highlighting hydrophobic regions and flanking charged residues that may influence ER targeting pathway selection. Their ability to insert into the ER membrane under co-translational conditions as well as whether transient translocation of their N-terminus across the ER membrane could be detected according to [42,45] (as described in (**C**)) is indicated. n.d.: not determined. (**C**) ERTOLD proteins AUP1, METTL7B, and HSD17B11 transiently expose their N-termini to the ER lumen before reaching the lipid droplet (LD) surface. To adopt the monotopic membrane topology required for LD association, two scenarios are possible: transient N-terminal exposure during insertion, followed by immediate hairpin formation (scenario 1), or initial bitopic insertion, with a subsequent topological shift occurring at a later timepoint, potentially assisted by additional factors (scenario 2). (**D**) Alternative ER insertion pathways for signal sequence containing APOE. Left: APOE follows an SRP-dependent route, undergoing signal peptide (in orange/coral red) cleavage by signal peptidase (SPase) and translocation into the ER lumen for incorporation into lumenal lipoproteins. Right: Under LD biogenesis conditions, APOE bypasses lumenal translocation and partitions to LD surfaces as a monotopic ERTOLD protein. (**E**) The ER-resident protein REEP5 shares a monotopic hairpin topology with many ERTOLD proteins and relies on SND pathway components for ER membrane insertion. SR, SRP receptor; HIG2, hypoxia-inducible gene 2 protein; METTL, methyltransferase-like protein; HSD17B, 17-beta-hydroxysteroid dehydrogenase; AUP1, ancient ubiquitous protein 1; RDH14, retinol dehydrogenase 14; DHRS3, short-chain dehydrogenase/reductase 3; UBXD8, UBX domain-containing protein 8; APOE, apolipoprotein E; REEP5, receptor expression-enhancing protein 5; SND, SRP-independent.

More recently, Leznicki et al. systematically investigated the ER targeting and membrane insertion requirements for a larger set of ERTOLD proteins employing cell-free translation systems and cell-based assays [45]. Based on the position of the hydrophobic membrane-embedded regions, some ERTOLD proteins are inserted into membranes co-translationally, while others are inserted post-translationally. Specifically, proteins with longer hydrophilic domains preceding the membrane-embedded segments, such as UBXD8 and HSD17B7, follow a post-translational route to the ER. In contrast, proteins with N-terminal positioning of the membrane-embedded hydrophobic regions (AUP1, HSD17B11, METTL7A, METTL7B, and HIG2) are co-translationally inserted into the ER membrane. Interestingly, the insertion of AUP1, METTL7B, and HSD17B11 requires the ER membrane complex (EMC) [45] (Figure 3A), a recently characterized multimeric membrane insertase related to the Oxa1/YidC/Alb3 family. EMC supports membrane integration during both co- and post-translational insertion of several ER membrane proteins and co-operates with the Sec61 translocon [28,59–64]. Accordingly, the ER insertion of AUP1, METTL7B, and HSD17B11 also involves the SR and Sec61, albeit independently of its translocation activity and potentially mediating ER targeting in conjunction with EMC [45].

Notably, the hydrophobic regions in oleosin and caveolin-1 are both centrally located within their sequences and significantly longer than the more N-terminally positioned hydrophobic regions of EMC-inserted clients studied by Leznicki et al. (Figure 3B). A unified framework in which TMDs with short flanking translocated segments are integrated by the EMC complex, while those with longer flanking regions rely on the Sec61 translocon was recently proposed [30]. This phenomenon could explain that oleosins and caveolin-1 employ a co-translational biosynthetic pathway that exclusively relies on Sec61 for membrane integration, whereas proteins with more N-terminal hairpin regions might instead depend on the collaborative action of Sec61 and EMC. However, it is conceivable that oleosins and caveolins also utilize the synergistic Sec61-EMC-mediated pathway, while the role of the EMC complex was missed in the earlier studies due to its unknown function at that time (Figure 3B). It will be interesting to test directly how structural differences influence co-translational ER targeting and membrane insertion of LD-destined proteins, as well as to decipher the involvement of the EMC in this process.

#### 2.1.2 Topological rearrangements of ER-inserted LD proteins

A monotopic topology is a hallmark feature of LD proteins, and most ERTOLD proteins that have been topologically investigated indeed adopt a monotopic topology in the ER bilayer as well as in the LD monolayer [16–18]. Interestingly, however, glycosylation-based *in vitro* reconstitution systems revealed that the co-translationally targeted proteins AUP1, METTL7B, and HSD17B7 translocate their N-termini into the ER lumen during membrane insertion [45]. Because transmembrane-spanning proteins are excluded from LD monolayers, the translocated N-termini are only transiently exposed to the ER lumen, and LD-destined proteins need to undergo a topological rearrangement when transitioning from a bilayer-spanning to a monotopic topology [45] (Figure 3C). Two potential mechanisms were proposed for the co-translational insertion of LD proteins via the EMC: the topological rearrangement required for LD localization could either occur during EMC-mediated membrane insertion (Figure 3C, scenario 1) or following initial membrane integration in a transmembrane-spanning topology (Figure 3C, scenario 2). Whether this rearrangement then occurs spontaneously or whether it requires the assistance of additional proteins remains unclear [45], but the latter case could provide means to spatially and/or temporally regulate the abundance of proteins that can partition to LDs. Stalled nascent chain or site-specific cross-linking *in vitro* experiments could shed light on the temporal framework underlying the topological switch and enable the identification of factors involved [65,66].

Translocation of LD-destined proteins across the ER membrane may not exclusively be a transient phenomenon. For example, fatty acyl-CoA reductase 1 (FAR1) adopts a type II membrane topology, in which its C-terminus has translocated across the membrane into the organelle lumen in the ER and on peroxisomes, both bilayer-encapsulated organelles. In contrast, and consistent with the requirement for a monotopic topology, both termini are exposed toward the cytosol when FAR1 is located on LDs [67]. Whether FAR1 undergoes a structural rearrangement during its partitioning to the LD surface or whether it can engage with different membrane targeting and insertion pathways during its biogenesis to establish distinct subpopulations remains to be investigated.

An example of the latter scenario is APOE, a secretory apolipoprotein that comprises an N-terminal signal sequence that mediates its co-translational SRP-dependent ER targeting and which can be cleaved off during translocation into the ER lumen [68]. More recently, however, it was shown that upon acute fatty acid loading of astrocytes, APOE can subvert translocation across the ER membrane, which results in a non-cleaved form of APOE that associates with the cytosolic side of the ER membrane, from where it can partition to the LD surface [69] (Figure 3D). The molecular details underlying this topological decision-making are unknown, but it is tempting to speculate that EMC contributes to alternative insertion fates of APOE, preventing signal sequence cleavage, and that lipid-mediated membrane parameters influence these processes. More systematic studies are required to decipher whether such alternative membrane targeting routes are dedicated to cell types that are capable of both secretion of lipoproteins and LD biogenesis and which metabolic and/or signaling statuses influence the fate of proteins like APOE.

### 2.2 SRP-independent ER targeting of LD-destined proteins and redundancy in targeting pathway selection

An SRP-independent (SND) co-translational ER targeting pathway has recently been identified in yeast [70]. In this system, Snd1 acts as the cytosolic mediator, escorting the newly synthesized substrate to its cognate heterodimeric ER membrane receptors, Snd2 and Snd3 (Figure 3E). Due to its overlapping substrate spectrum with other ER targeting pathways, the SND system is considered to play a compensatory role, stepping in to facilitate protein targeting when other pathways are impaired to ensure fidelity in ER targeting, e.g. during stress adaptation [59,70–73].

The transmembrane proteins TMEM208 [71,74] and TMEM109 [75] were identified as human homologs of Snd2 and Snd3, respectively, whereas a functional homolog for Snd1 has yet to be identified in higher eukaryotes. Although the SND pathway can accommodate substrates with signal sequences at their N-terminus, it exhibits a preference for cargos containing centrally located or even C-terminal TMDs with lower hydrophobicity [70], a characteristic shared by many ERTOLD proteins, rendering them potential SND substrates. Interestingly, depletion of either TMEM208 or TMEM109 led to decreased ER insertion of REEP5, which is a monotopic hairpin protein whose reticulon homology domain (RHD) contains two consecutive hairpins closely mirroring the structural features of hairpin-containing ERTOLD proteins and imposing comparable constraints on membrane insertion [75] (Figure 3E). In contrast with ERTOLD proteins, however, REEP5 is an ER-resident protein and does not partition to LDs. It will be interesting to test whether LD-destined hairpin proteins can also engage with and potentially even rely on the SND system. Notably, TMEM208 was very recently characterized as a factor that accelerates substrate handover from SRP to the downstream translocation/insertion machinery, which appears especially crucial for ensuring ER insertion fidelity of rather hydrophobic substrates and multipass transmembrane proteins [76]. Thus, at least eukaryotic Snd2 can contribute to SRP-mediated ER targeting, a completely unexplored component in the coupling of targeting and membrane insertion of LD-destined hairpin proteins.

### 2.3 TRC/GET-mediated ER targeting of LD-destined proteins

Not all proteins can be co-translationally inserted into the ER membrane: tail-anchored (TA) proteins contain a single TMD at their extreme C-terminus, which conveys the targeting information for the TRC/GET pathway and which only emerges from the ribosome after completion of translation [28,77–80]. TA proteins are captured by pre-targeting complexes composed of several cascading cytosolic chaperones and are then post-translationally transferred to the soluble targeting factor TRC40 (Get3 in yeast), which then associates with the ER membrane-bound receptors WRB and CAML (Get1/2 in yeast) [78,81–83]. The position of the hydrophobic targeting sequence is considered decisive for ER targeting pathway selection during protein biogenesis, with more N-terminal sequences favoring the co-translational SRP system, whereas C-terminal TMDs engage with the TRC/GET pathway (Figure 2) [70].

**Figure 4:**
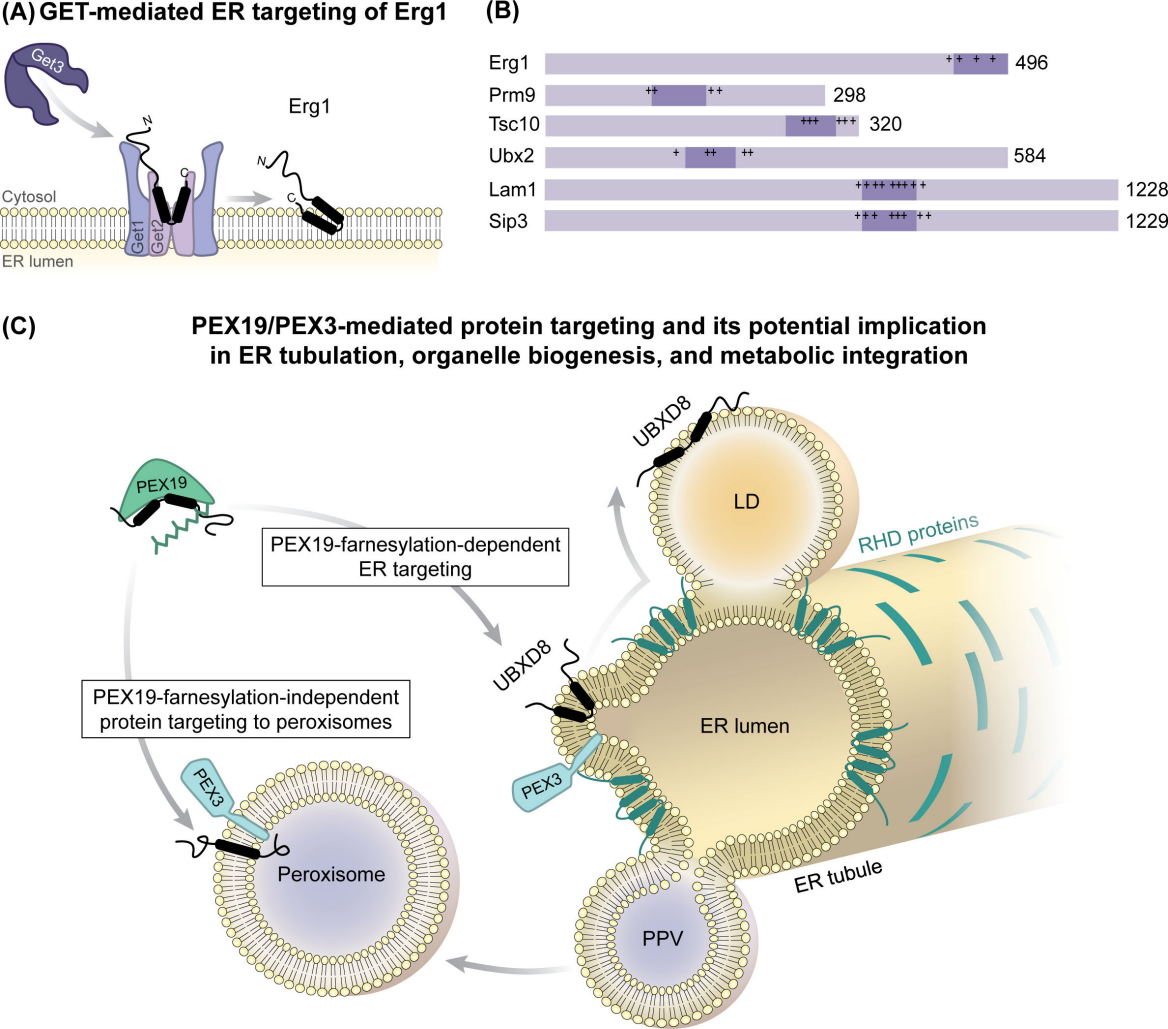
Post-translational ER membrane insertion of ERTOLD proteins. (**A**) Guided entry of tail-anchored (GET)-mediated post-translational insertion of yeast Erg1 into the endoplasmic reticulum (ER) membrane. (**B**) Domain organization of yeast proteins with putative hairpin regions that require Get3 for ER membrane insertion. Hydrophobic segments and flanking charged residues are highlighted, indicating that C-terminal positioning of hydrophobic regions is not strictly required for post-translational Get3-dependent insertion. (**C**) PEX19 and PEX3, central mediators of peroxisomal protein targeting, also recruit the ERTOLD protein UBXD8 post-translationally to discrete ER membrane domains adjacent to peroxisomes. RTN4C and ARL6IP, members of the ER-shaping reticulon homology domain (RHD) protein family, similarly require PEX19 for ER targeting but remain ER-resident, while UBXD8 can partition to lipid droplet (LD) monolayers. Several RHD proteins localize to ER regions involved in LD and pre-peroxisomal vesicles (PPVs) formation, potentially within the same domain. Since LDs and peroxisomes collaborate in lipid metabolism, co-ordinating organelle biogenesis with targeted delivery of implicated proteins may provide functional advantages. Farnesylation of PEX19 is critical for the ER/LD targeting of UBXD8, whereas peroxisome biogenesis from the ER can occur independently. Consistently, disrupting PEX19 farnesylation alters LD turnover, highlighting a mechanistic, peroxisome-unrelated role of PEX19 in LD dynamics. Erg1, ergosterol biosynthetic protein 1; Prm9, pheromone-regulated membrane protein 9; Tsc10, temperature-sensitive CSG2 suppressor protein 10; Ubx2, UBX domain-containing protein 2; Lam1, lipid transfer protein anchored at membrane contact sites 1; Sip3, SNF1-interacting protein 3; PEX, peroxin; UBXD8, UBX domain-containing protein 8; RTN4C, reticulon-4; ARL6IP, ADP-ribosylation factor-like protein 6-interacting protein 1

However, the yeast ERTOLD protein Erg1 is an atypical client of the GET pathway [84] (Figure 4A). While it harbors a hydrophobic hairpin region at its extreme C-terminus, this C-terminal positioning is not a prerequisite for engaging with the GET pathway because C-terminal fusion with GFP does not alter GET-dependency of Erg1 (Figure 4B). Apart from Erg1, five additional candidates, identified through a bioinformatic screen based on predicted internal hairpin-like regions, showed GET-dependent ER localization. Among these, Tsc10 and Ubx2 have been experimentally validated as monotopic hairpin ERTOLD proteins [4,85–87]. Since the predicted hairpin regions in these proteins are not confined to the C-termini [84], the GET pathway may recognize a broader client spectrum beyond canonical TA proteins, including monotopic hairpin-containing proteins with variable positioning of their membrane-embedded regions. Mechanistically, Get3 may recognize long hydrophobic hairpins differently from canonical TA proteins. Both helices of the Erg1 hairpin contribute to Get3 binding [84], raising the possibility that multimeric forms of Get3 [79,88–91] may be required to capture hairpin-containing proteins, allowing accommodation of both helices and potentially alleviating the requirement for C-terminal positioning.

Whether these concepts are conserved in the mammalian TRC pathway remains speculative, as no mammalian ERTOLD clients have been identified to date. The mammalian Erg1 homolog squalene monooxygenase (SQLE) differs from yeast Erg1 by an N-terminal sterol-sensing domain [92]. In yeast, both full-length and N-terminally truncated SQLE can substitute for Erg1, but only the truncated form shows Get3 dependence and LD localization, suggesting that the N-terminus may override TRC/GET-mediated insertion [84]. In mammals, both variants exist due to partial proteolytic degradation of full-length SQLE [93]. Whether these forms follow distinct ER targeting routes remains unclear, but if confirmed, it would suggest that proteolytic processing adds regulatory complexity to pathway selection.

Other factors that could determine whether ERTOLD proteins engage the GET/TRC pathway remain largely unexplored, but the hydrophobicity of membrane-embedded regions could play a role: TRC40 preferentially mediates insertion of TA proteins with high hydrophobicity [28,30,77], while TA proteins of lower hydrophobicity rather employ cytosolic chaperones (e.g. HSP70) and the EMC for their membrane integration [59]. Systematic mutational studies could reveal the determinants for ER targeting pathway selection of ERTOLD proteins, including the role of hydrophobicity and charge distribution [34]. Redundancy with alternative targeting pathways, however, could mask the effects of Get3/TRC40 loss under steady-state conditions. The GET pathway becomes critical when cells require rapid up-regulation of specific clients, such as Erg1 during sterol depletion [84]. Therefore, future efforts to define client spectra should consider not only substrate identity but also expression kinetics and cellular demand, as high-fidelity targeting is particularly important under conditions that require rapid adaptation through acutely increased protein biogenesis.

### 2.4 PEX19/PEX3-mediated ER targeting of LD-destined proteins and its implication in ER-derived organelle biogenesis

ERTOLD proteins must comply with two requirements: First, they require targeting information, ensuring specific insertion into the ER membrane and preventing mislocalization to other organelles. Second, as this information is encoded within their hydrophobic hairpin region, it must be compatible with a monotopic topology, which eventually allows bilayer-to-monolayer partitioning. In addition, ERTOLD proteins likely encompass additional intrinsic features for regulated partitioning to LDs. Thus, accommodating all these characteristics likely renders ERTOLD proteins susceptible to different ER targeting pathways, and one might wonder whether selection of one specific ER targeting pathway is at all relevant for the physiological function of the proteins.

Evidence for the importance of which ER targeting pathway is employed stems from the discovery of an unconventional ER targeting mechanism for the ERTOLD protein UBXD8 [15,47,53,54]. Unexpectedly, the post-translational ER insertion of UBXD8 relies on two peroxisomal biogenesis factors: the cytosolic chaperone PEX19 and its membrane-bound receptor PEX3 [53]**,** both originally found to mediate protein insertion into peroxisomal membranes [94–98]. Upon UBXD8 synthesis in the cytosol, soluble PEX19 binds to its hydrophobic region and post-translationally directs it to specific subdomains of the ER membrane in a PEX3-dependent process (Figure 4C). Notably, farnesylation of PEX19 ensures the selective insertion of UBXD8 into the ER from where it can subsequently partition to the LD monolayer [53]. Quantitative proteomics revealed that in addition to UBXD8, PLIN5, RDH10, and RAB4A are depleted from LDs when only a farnesylation-deficient variant of PEX19 was expressed in PEX19 knock-out cells [99]. Whether these PEX19-dependent LD proteins also directly employ PEX19/PEX3 or whether their compromised LD localization is a downstream consequence of altered LD properties remains to be investigated. Under these conditions, however, cells exhibit a lipogenic phenotype with excessive triglyceride storage in LDs that cannot be fully metabolized under catabolic conditions, while peroxisome biogenesis and function are restored [99].

Further evidence for the importance of ER targeting pathway selection stems from the discovery that RTN4C and ARL6IP, two monotopic ER-resident RHD-containing proteins, also rely on PEX3/PEX19 for their insertion into discrete ER subdomains [100]. RHD-containing proteins promote tubulation of the ER by stabilizing membrane curvature [101] (Figure 4C). Consequently, their post-translational insertion has been proposed to functionally couple ER tubulation with peroxisomal biogenesis from the ER membrane [100]. Likewise, LD biogenesis preferentially occurs in tubulated ER regions [102–104], and evidence suggests that peroxisomes and LDs may emerge from the same specialized subdomains within the ER that are marked by RHD-containing proteins [Pex30 in yeast and its homolog multiple C2 and transmembrane domain-containing protein 2 (MCTP2) in mammalian cells] [105,106] (Figure 4C). Indeed, when inserted into semi-permeabilized cells, newly synthesized UBXD8 accumulates in distinct subdomains along ER tubules that are often in close proximity to peroxisomes [53].

The ER is a heterogeneous network with different morphologies and functional specializations [107]. ER tubules are usually devoid of ribosomes and therefore part of the smooth ER, which is presumably specialized in lipid metabolic pathways rather than protein biogenesis. It is therefore tempting to speculate that post-translational insertion of proteins, which would disrupt the rough ER sheets by inducing curvature or which are involved in the biogenesis of lipid metabolic organelles from the ER membrane, is mechanistically separated from bulk ER protein insertion. Together, these two examples suggest that dedicated ER targeting pathway selection can be pivotal beyond simply inserting proteins into the ER with a broader downstream role in controlling organelle biogenesis and inter-organelle communication in lipid metabolism.

## 3. Outlook: current challenges in decoding ER targeting pathway selection for ERTOLD proteins

Unlike transmembrane-spanning proteins, LD-destined monotopic proteins represent a relatively unexplored class with respect to their biogenesis, including selective ER targeting and topogenesis. The examples outlined above, however, suggest that the different ER targeting pathways may introduce regulatory checkpoints in cellular lipid metabolism. To advance this field, we identify three key challenges that should be addressed:

### Defining protein-intrinsic determinants for pathway selection

For transmembrane-spanning proteins, the length, positioning, and hydrophobicity of their membrane-embedded domains, as well as the presence of membrane-flanking charges, can affect ER targeting pathway selection. Potentially, similar properties influence pathway selection of ERTOLD proteins. Due to their unique hairpin-type topology, however, ERTOLD proteins possess additional features ensuring their correct membrane topology and introducing additional complexity. Indeed, mutation of, for example, the central kink-inducing proline motifs or membrane-flanking positive charges to challenge ER pathway selection can disrupt protein topogenesis, converting monotopic proteins into transmembrane forms or impairing LD localization [17,55,108–110]. Therefore, identifying mutants that preserve correct topology and LD targeting will be essential for dissecting intrinsic features that determine ER targeting pathway selection. Molecular dynamics simulations offer a promising strategy to predict such features, which can then be validated experimentally.

### Pathway redundancy and metabolic modulation

ERTOLD proteins may engage multiple ER targeting pathways, with selection influenced by metabolic state or tissue-specific signals. The SND pathway could play a context-dependent role, especially under stress or in specialized cell types. A major challenge lies in identifying *bona fide* ERTOLD cargos of SND, given the overlapping substrate spectra among targeting pathways and the non-essential nature of SND components, which complicates dependency testing.

Likewise, the context-dependent membrane integration of, e.g. APOE may diverge between tissues or signaling cues. Whether this reflects transient engagement with multiple pathways or a regulated switch in topology remains an open question. Understanding the molecular logic behind such plasticity, including the role of targeting and membrane insertion factors, or post-translational modifications, could illuminate broader principles of ERTOLD protein biogenesis and function. To dissect this complexity, systematic screening of ERTOLD proteins for pathway engagement under specific metabolic or developmental conditions is needed, ideally using combinatorial knockdowns, proximity labeling, and topology-sensitive reporters.

### Unassisted membrane association and annotation gaps

Finally, the question arises whether those ERTOLD proteins that are not inserted into the ER membrane with hydrophobic hairpin motifs but stably associate with the membrane via interfacial amphipathic helices, such as DHRS3 [21], rely on any dedicated ER targeting machinery or whether they could spontaneously engage with the phospholipid bilayer. At least DHRS3 can post-translationally insert into ER membranes [45], but whether lipid-mediated features of the ER membrane alone are sufficient for selective ER recruitment and for preventing mislocalization to, e.g. mitochondria needs to be tested. Such a scenario, however, would raise the question of how direct insertion of DHRS3 into the LD monolayer is prevented. Similarly, proteins formerly annotated as CYTOLD proteins, such as hallmark LD proteins of the PLIN family, were recently shown to localize to the ER as well and are therefore *bona fide* ERTOLD proteins [14,111,112]. PLIN3 can associate with diglyceride-enriched ER membranes [111], potentially explaining preferential binding to the ER over LDs. For ER-localized PLIN1 [14,112], however, it remains to be investigated whether a dedicated ER targeting machinery is employed.

Together, these challenges highlight the need for integrative approaches combining structural biology, cell biology, and computational modeling to uncover the molecular logic of ER targeting pathway selection and to decode how cells fine-tune LD protein targeting in response to physiological demands. Addressing them will not only refine our understanding of LD protein biogenesis but may also reveal new therapeutic entry points for metabolic diseases.

**Perspectives**

- Lipid droplet (LD)-localized proteins play a central role in lipid metabolism and are increasingly linked to major diseases such as obesity, cardiovascular disorders, and cancer. It is, therefore, critical to unravel the molecular basis of LD protein biogenesis, including their initial targeting to the endoplasmic reticulum (ER) membrane before they partition to the LD surface.
- Emerging evidence suggests that the choice of ER targeting and insertion pathways can shape the fate and function of LD-destined proteins and influence the formation and identity of ER-derived organelles.
- Future efforts should focus on systematically identifying the protein-intrinsic signals and cellular contexts that govern ER targeting pathway selection, while refining the classification of ERTOLD proteins to better reflect their dynamic roles across tissues and metabolic states.

## Abbreviations

ANKRD22: ankyrin repeat domain-containing protein 22
APOE: apolipoprotein E
ARL6IP: ADP-ribosylation factor-like protein 6-interacting protein 1
AUP1: ancient ubiquitous protein 1
CTS: C-terminal segment
DHRS3: short-chain dehydrogenase/reductase 3
EMC: ER membrane complex
ER: endoplasmic reticulum
Erg1: ergosterol biosynthetic protein 1
FAR1: fatty acyl-CoA reductase 1
GET: guided entry of tail-anchored
HIG2: hypoxia-inducible gene 2 protein
HMD: hydrophobic membrane domain
HSD17B: 17-beta-hydroxysteroid dehydrogenase
LD: lipid droplet
Lam1: lipid transfer protein anchored at membrane contact sites 1
MCTP2: multiple C2 and transmembrane domain-containing protein 2
METTL: methyltransferase-like protein
PEX: peroxin
PLIN: perilipin
PPVs: pre-peroxisomal vesicles
Prm9: pheromone-regulated membrane protein 9
RDH14: retinol dehydrogenase 14
REEP5: receptor expression-enhancing protein 5
RHD: reticulon homology domain
RTN4C: reticulon-4 C
SND: SRP-independent
SPase: signal peptidase
SQLE: squalene monooxygenase
SR: SRP-receptor
SRP: signal recognition particle
Sip3: SNF1-interacting protein 3
TA: tail anchored
TMD: transmembrane domain
TRC: transmembrane recognition complex
Tsc10: temperature-sensitive CSG2 suppressor protein 10
UBXD8: UBX domain-containing protein 8
Ubx2: UBX domain-containing protein 2
VCP: valosin-containing protein
iMS: integral membrane segment
